# A Peculiar Case of the Abscopal Effect: Radioactive Iodine Therapy Incidentally Palliating Marginal Zone Lymphoma

**DOI:** 10.1155/2015/281729

**Published:** 2015-02-02

**Authors:** Robert C. Kornas, Sarah-Kim Shields, Lyle S. Goldman

**Affiliations:** ^1^Providence Hospital and Medical Centers, 16001 W. Nine Mile Road, Suite 401, Southfield, MI 48075, USA; ^2^St. Joseph Mercy Oakland Hospital, 44405 Woodward Avenue, Pontiac, MI 48341, USA

## Abstract

The abscopal effect is an extremely rare phenomenon occurring when irradiation or treatment of a primary tumor burden not only results in debulking of the targeted site but also reduces tumor size at distant sites from the intended treatment area. We present the abscopal effect occurring in a patient with low-grade marginal zone lymphoma who subsequently received radioactive iodine therapy for papillary thyroid carcinoma. She was 67 years old when a routine complete blood count at her primary care physician's office yielded a persistent leukocytosis of 14,500/*μ*L with lymphocytosis of 9,870/*μ*L. Immunophenotyping and fluorescence in situ hybridization (FISH) analysis confirmed low-grade marginal zone lymphoma. Over eight years, her peak leukocyte and lymphocyte counts were 24,100/*μ*L and 18,100/*μ*L, respectively. Subsequently, she was diagnosed with papillary thyroid carcinoma after presenting with a new complaint of dysphagia. A total thyroidectomy was performed, followed by 172.1 millicuries of oral I-131 sodium iodine radioactive ablation therapy. Following treatment, her leukocyte and lymphocyte counts were 3,100/*μ*L and 1,100/*μ*L, respectively. Over the next four years, her leukocyte and lymphocyte counts remained within normal limits and she remained symptom free. To our knowledge, there has never been a published report describing the use of radioactive iodine causing abscopal effect benefits for patients with underlying lymphoproliferative diseases.

## 1. Introduction

The abscopal effect is an extremely rare phenomenon that occurs when localized irradiation or treatment of tumor not only results in debulking of the targeted site but also reduces tumor size at sites that are distant from the intended treatment area. The overall effect on the tumor burden can be remarkably profound, with documented cases of previously malignant growths resolving from this indirect irradiation and treatment.

Albeit rare, cases of the abscopal effect have been described in a variety of cancers, including lymphoma, papillary adenocarcinoma, melanoma, chronic lymphocytic leukemia, and hepatocellular carcinoma [1–5]. At the time of this publication, the vast majority of documented cases of the abscopal effect have been witnessed in external beam radiation therapy. This case report demonstrates the manifestation of the abscopal effect occurring in a patient initially presenting with low-grade marginal zone lymphoma who subsequently received radioactive iodine (RAI) therapy for papillary thyroid carcinoma.

## 2. Case Presentation

A 67-year-old female patient of Ukrainian descent initially presented after a routine complete blood count (CBC) at her primary care physician's office with leukocytosis with an elevated lymphocyte count. Subsequent CBC six months later showed a persistently elevated leukocyte count of 14,500/*μ*L with lymphocytosis of 9,870/*μ*L. For this reason, she was referred for further evaluation, workup, and management.

At the time of evaluation, the patient was entirely asymptomatic. She denied weight loss, fever, and night sweats. She denied lymphadenopathy, cough, nausea, abdominal discomfort, changes in bowel habits, or skeletal discomfort. Her only significant past medical history included hypertension and hypercholesterolemia. However, she did have significant radiation exposure from the Chernobyl nuclear reactor disaster in 1986 while she was living in Ukraine. Her family history was unremarkable for cancer. Physical examination was unremarkable except for a one-centimeter (cm), nontender right axillary lymph node. Otherwise, she did not have additional lymphadenopathy, thyromegaly, hepatosplenomegaly, or peripheral edema. A repeat CBC performed in the office showed a hemoglobin of 13.1 g/dL, a hematocrit of 39%, a platelet count of 251,000/*μ*L, and a leukocyte count of 15,300/*μ*L with 10,400/*μ*L lymphocytes. A peripheral blood smear was performed which showed evidence of lymphocytosis composed of small to intermediate sized lymphocytes with irregular nuclear contours and occasional clefted nuclei without the presence of discrete nucleoli. Red cell, platelet, and granulocyte morphology was normal.

Furthermore, immunophenotyping revealed monoclonal B cell lymphocytosis composed of intermediate sized lymphocytes and irregular nuclei. The lymphocytes were lambda light chain positive; in addition, CD19 and CD20 were also positive. CD5 and CD10 were negative. Fluorescence in situ hybridization (FISH) analysis indicated positivity for trisomy 12 in 57% of the cells, p53 deletion in 10% of the cells, and IgH gene deletion in 6.5% of the cells (Figure 2). She was diagnosed with B cell lymphoproliferative process of marginal zone origin.

Computed tomography (CT) imaging of the chest, abdomen, and pelvis was performed to assess adenopathy and splenomegaly. There was no evidence of axillary or hilar lymphadenopathy; however, there was a soft tissue mass measuring 2.0 centimeters (cm) identified in the retroesophageal space, which caused anterior displacement of the esophagus, consistent with adenopathy. To further investigate this mass, an esophagram was performed which showed no evidence of reflux or mucosal abnormalities in the distal esophagus. Roughly six months later, a repeat CT scan of the thorax was ordered to follow up on the 2.0 cm mass. In comparison to the prior study, there was no evidence of the mass posterior to the esophagus as had been previously identified. Due to the fact that the patient did not have any symptoms consistent with marginal zone lymphoma and her CBC and clinical examination were stable, cytoreductive therapy was not indicated. The patient was monitored with follow-up CT scans of the thorax and routine CBCs.

From the initial presentation, the patient underwent annual CT scans of the thorax to find out any evidence of progression of her lymphadenopathy. Over the course of approximately eight years, the only significant CT findings included a 1.5 cm × 2.0 cm left axillary lymph node that was identified but regressed over the course of one year. Additionally, mild splenomegaly was noted, measuring 13 cm in length, which remained stable in size. No additional lymphadenopathy was identified and she remained asymptomatic. During this eight-year timeframe, her leukocyte and lymphocyte counts were monitored biannually. The patient's peak and nadir leukocyte counts were 24,100/*μ*L and 11,200/*μ*L, respectively, with a maximum lymphocyte count of 18,100/*μ*L and nadir of 7,000/*μ*L.

Nearly nine years after the initial diagnosis of marginal zone lymphoma and careful monitoring of leukocyte and lymphocyte counts, the patient presented with complaints of dysphagia and persistent sore throat for three weeks. A CT scan of the neck and thorax was ordered and showed evidence of a mass within the thyroid gland (Figure 3(a)). Subsequent ultrasound (Figure 3(b)) and fine needle aspiration ultrasound guided biopsy revealed papillary thyroid carcinoma. She underwent a total thyroidectomy and was treated with 172.1 millicuries (mCi) of oral I-131 sodium iodine radioactive ablation therapy. She responded favorably to treatment and had no complications. Postthyroidectomy ultrasound showed no evidence of residual thyroid tissue.

**Figure 1:**
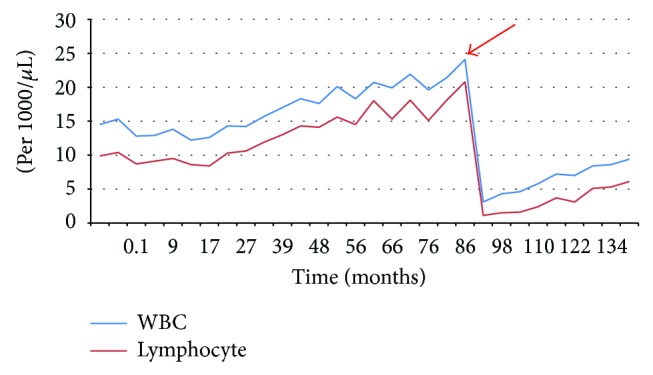
Pre- and postradioactive iodine therapy leukocyte and lymphocyte counts. The figure displays leukocyte and lymphocyte counts (per *μ*L) over 12-year (144 months) timeframe. Red arrow correlates to the initial dose of radioactive iodine therapy for papillary thyroid carcinoma and signifies the drop in leukocyte count from 24,100/*μ*L to 3,100/*μ*L and in lymphocyte count from 18,100/*μ*L to 1,100/*μ*L.

**Figure 2:**
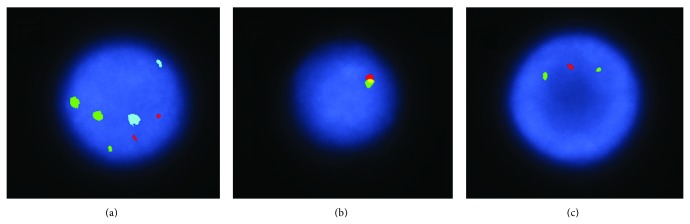
Fluorescence in situ hybridization (FISH) analysis. The fluorescence in situ hybridization (FISH) study for CLL was abnormal. A multiprobe panel consisting of an ATM gene at 11q22.3, a chromosome 12 pericentromeric DNA, a chromosome 13q14 putative tumor suppressor gene probe, an IgH gene, and a p53 chromosome 17 DNA probe revealed deletions of IgH and p53 genes and a trisomy for chromosome 12. Deletion of p53 is correlated with a poor prognosis. Chromosome 11 FISH results were normal. (a) Alpha 12 and 13q14 DNA probe: CEP 12 = green; 13q14 (D13S319) = red; 13q34 = aqua. (b) LSI IgH (14q32.3) Dual Color Break Apart DNA probe: normal IgH locus = yellow and side-by-side red-green. (c) ATM and p53 DNA probe: ATM (11q22.3) = green; p53 (17p13) = red.

**Figure 3:**
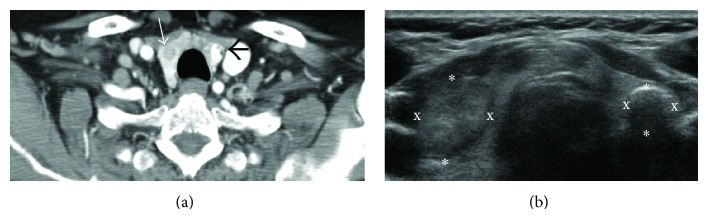
(a) Contrast enhanced computed tomography (CT) of the thorax. Thin, white arrow indicates the largest nodule in the mid pole of the right thyroid lobe, measuring 1.6 × 1.2 × 1.4 cm. Black, thick arrow demonstrates a calcified nodule in the mid-to-lower left thyroid lobe measuring 1.1 × 0.9 × 0.9 cm. There are multiple scattered calcified nodules noted throughout the thyroid gland. (b) Axial ultrasound of the thyroid gland. There is evidence of a solid isoechoic nodule in the right thyroid lobe measuring 1.6 × 1.2 × 1.4 cm and a peripherally calcified nodule in the left thyroid lobe measuring 1.1 × 0.9 × 0.9 cm.

One month prior to her treatment with RAI, the patient's measured leukocyte count was 24,100/*μ*L with 18,100/*μ*L lymphocytes and she was without B-symptoms on clinical examination. After the patient was treated for papillary thyroid carcinoma, she was reevaluated six months later with routine CBC to follow up and monitor her previously diagnosed marginal zone lymphoma. Not only was she asymptomatic but also her leukocyte count was now 3,100/*μ*L and her lymphocyte count was 1,100/*μ*L. Over the course of the next four years, her leukocyte and lymphocyte counts were trended and monitored at six-month intervals. At one year after RAI therapy, the patient showed a leukocyte count of 4,300/*μ*L and a lymphocyte count of 1,500/*μ*L. At two years after RAI therapy, her leukocyte count was 5,800/*μ*L and lymphocyte count was 2,400/*μ*L; three-year leukocyte count was 7,000/*μ*L and lymphocyte count was 3,100/*μ*L; and four-year leukocyte count was 8,600/*μ*L with 5,300/*μ*L lymphocytes (Figure 1).

The patient has continued to follow up and has had biannual CBC laboratory analysis to trend her leukocyte and lymphocyte counts. Although her levels have slowly started to increase over the four-year period following her RAI treatment, she has remained asymptomatic and has still not required cytoreductive therapy.

## 3. Discussion

As previously documented and reported, radiotherapy, albeit rare, has been shown to have significant impact on distant tumor burden other than the targeted area of treatment. Typically seen in cases involving external beam ionizing radiation, to our knowledge, there has never been a published report describing the use of radioactive iodine resulting in abscopal effect benefits for patients with underlying lymphoproliferative diseases.

Although the exact mechanism by which the abscopal effect occurs is unknown, there have been proposed theories regarding the abscopal antitumor effect. The first theory applies to leukemia and lymphomas. During splenic irradiation, diseased lymphocytes circulate through the irradiated spleen and as the splenic size decreases, the systemic masses also drastically decrease in size due to the irradiated lymphocyte effect on those sites [6–8]. The second hypothesis applies to solid tumors. Through the release of cytokines and mitotic inhibitors caused by local irradiation treatment, an antitumor effect is mediated and distant sites of disease are acted upon by these cytokines [4]. The final theory is that the abscopal effect is mediated by the immune system. Tumor irradiation releases inflammatory factors, such as natural killer cells, that may augment the immune system against malignant lesions expressing similar tumor antigens [2].

In our patient with marginal zone lymphoma, treatment with radioactive iodine therapy for a subsequently discovered papillary thyroid carcinoma resulted in a drastic improvement in her leukocyte and lymphocyte counts. The use of radioactive iodine resulted in unexpected cytoreduction therapy for her marginal zone lymphoma. The uniqueness of the case, in addition to four years of laboratory values available following treatment, is an excellent example of the abscopal effect which is the first to be described in a case of radioactive iodine leading to unintended treatment for low-grade marginal zone lymphoma.
